# Nano-scale depth-varying recrystallization of oblique Ar^+^ sputtered Si(111) layers

**DOI:** 10.1038/s41598-020-68873-8

**Published:** 2020-07-17

**Authors:** Divya Gupta, G. R. Umapathy, Rahul Singhal, Sunil Ojha, Sanjeev Aggarwal

**Affiliations:** 1 Department of Physics, Kurukshetra University, Kurukshetra, 136119 India; 2 Inter University Accelerator Center, Aruna Asaf Ali Marg, New Delhi, 110067 India; 3 Malviya National Institute of Technology (MNIT), Jaipur, Rajasthan 302017 India

**Keywords:** Materials science, Nanoscale materials, Structural properties

## Abstract

Silicon, the workhorse of semiconductor industry, is being exploited for various functional applications in numerous fields of nanotechnology. In this paper, we report the fabrication of depth controllable amorphous silicon (a-Si) layers under 80 keV Ar^+^ ion sputtering at off-normal ion incidences of 30°, 40° and 50° and crystallization of these amorphous Si(111) layers under thermal annealing. We find that the irradiated samples were not fully amorphized even for the lowest oblique incidence of 30°. Sputtering at off-normal incidences induces depth controllable surface amorphization in Si(111). Annealing at temperature of 1,073 K is characterized by formation of depth-varying buried amorphous layer due to defect recrystallization and damage recovery. Some remnant tensile stress has been observed for recrystallized samples even for lowest oblique incidence. The correlation of amorphization and stress due to sputtering induced by oblique incidence has been discussed systematically. The possible mechanism of recrystallization is discussed in terms of vacancies produced in sputtering dominated regime and their migration during annealing treatment. Our results reveal that with appropriate selection of oblique ion beam sputtering parameters, depth controllable surface amorphization and recrystallization may be fine-tuned to achieve co-existing amorphous and crystalline phases, playing a crucial role in fabrication of substrates for IC industry.

## Introduction

Silicon, the workhorse of semiconductor industry, is being exploited for the various applications in numerous fields of nanotechnology like development of FETs and infrared detectors^1–3^. The combined amorphous and crystalline phases of silicon play a crucial role in the fabrication of substrates for IC industry and hence, deciding circuit performance and reliability^4–6^. The process of ion implantation has become a versatile tool for producing doped regions in FETs source, channel and drain in semiconductors^1,4–8^. In this regard, low energy ion irradiation is widely employed in IC industry for producing bipolar transistors in semiconductors, especially in silicon substrates. This technique offers advantage of implanting controllable quantity of incident ions into the shallow surface layers of substrate with good accuracy^5–8^.

In addition to these beneficial features, this non-equilibrium method disorders the equilibrium crystalline structure of the single crystal material by inducing amorphization as well as damage in the lattice^5–7,9,10^. The extent and nature of these ion beam induced imperfections in the lattice limits the performance and reliability of semiconductor devices and circuits^1,6,11^. This, in turn, necessitates the recovery of crystalline order of the lattice^5,9,10,12,13^. It is well known that recovery from disordered surface layers is eventually a diffusive process. Hence, the well known viable tool for restoring ordered crystalline structure, recovery of ion beam induced imperfections & defects and activation of dopants is thermal annealing^4,9,10,12^.

Ion implantation induced disorder and recovery processes have been reported by many researchers in literature^4,10–26^. In addition, implantation induced defects and its annealing has also been studied in different orientations of silicon such as Si(100)^4,11–13,21,22^, Si(110)^4,12^, Si(001)^23^ and the dependence of recrystallization behavior on electronic energy loss was reported. These studies have reported the amorphization and hence recrystallization behavior at normal incidences. Nakata et al^4,12^ have studied the recrystallization in Si for annealing temperatures ranging from 120 to 1,400 °C. Sahoo et al.^13^ have discussed the effect of annealing temperature on degree of recrystallization. Partial to complete recrystallization has been observed by varying the annealing temperature from 473 to 623 K. Turos et al.^24^ have performed the annealing treatment at temperatures of 650, 720 and 800 °C. Besides numerous technological applications, there are few relevant studies on the investigations of annealing behavior of Si(111) due to low energy ion implantations, e.g. Turos et al.^24^ investigated the disorder structure in neon implanted Si(111) after annealing. Labbani et al.^25,26^ investigated the surface damage induced in silicon and its recovery with respect to antimony dose and rapid thermal processing at 1,173 K.

None of the researcher attempted to address ion irradiation induced amorphization and defects and then its recrystallization behavior in Si(111) under the oblique incidences where the process of sputtering govern the degree of modifications. However, till date, there is no direct evidence in the existing literature regarding regrowth of amorphous Si layers in the sputtering dominating regime. This strongly motivated us to quantitatively address the recrystallization characteristics in amorphous Si(111) layers under off-normal argon ion sputtering. It will be more interesting to study Si(111) for amorphization and subsequent recrystallization under off-normal argon ion sputtering as it offers highest atomic density among all planar orientations of silicon. Further, Si(111) are ideal substrates for surface reconstruction, heteroepitaxial growth techniques, optoelectronic devices and generation of nano-electronics^27^. Hence, present research focuses on experimentally investigating the role of sputtering in regrowth of amorphous Si layers produced by oblique argon ion sputtering under thermal annealing. Brown et al^28,29^ have investigated the morphological evolution of Si(111) (n-type, As doped) during low energy (250–1,200 eV) irradiation at oblique incidence of 60° keeping the samples at temperatures between 500 and 750 °C. Due to very low energy of eV at constant off-normal incidence of 60° with variable fluence and simultaneous substrate heating, they observed the formation of rich variety of self-assembled array of nano-structures. They have mentioned that at these temperatures, recrystallization occurs faster than amorphization during single ion impacts; hence, they have not observed the formation of amorphous layers and its recrystallization. But the goal of present studies is to investigate the role of sputtering in amorphization and recrystallization behavior of Si(111). So, the oblique incidences have been varied from 50° to 30° at energy of 80 keV keeping ion fluence constant and thereafter annealing treatment at 1,073 K (800 °C).

Generally, incident ions at low energy interact with target atoms via nuclear energy loss^5,6^. This process of energy deposition creates a cascade of elastic collisions and displaces the target atoms from their initial sites and thus results in permanent atomic scale defects in the lattice^3–5^. In case of low energy and obliquely incident ion beam, incident ions produce a specific region of disorder along its path and lose its energy in this region of disorder in surface layers only. So, the extent of disorder and induced amorphization is confined majorly to the surface layers due to limited projected range of the incoming ions^3,5–7^.

Therefore, we have made an attempt to investigate the recrystallization behavior of surface region of Si(111) initially sputtered with 80 keV Ar^+^ ions under off-normal incidences of 30°, 40° and 50°. The recrystallization kinetics of these pre-amorphized Si(111) samples is reported under thermal annealing. An important practical consequence of our study is related to understanding of the recrystallization kinetics of amorphized Si produced under oblique and low argon beam irradiation.

## Experimental details

Single crystalline single side polished silicon wafers with (111) orientation were used as substrates. Amorphous surface layers were produced by sputtering with 80 keV Ar^+^ ions at off-normal incidences of 30°, 40° and 50°. For all the oblique incidences, a fixed ion dose of 3 × 10^17^ Ar^+^cm^−2^ at beam current density of ~ 3 μA cm^−2^ was used. 200 kV Ion Accelerator facility available at Ion Beam Centre, Kurukshetra University, Kurukshetra, India was used for carrying out sputtering on the silicon samples under a vacuum of ~ 2 × 10^–6^ Torr.

For defect annealing and recrystallization, each sample was annealed in oxygen atmosphere at a definite temperature of 1,073 K for 30 min. This thermal annealing process, in turn, formed a thin layer of SiO_2_ oxide over the sample surface which was expected to prevent the loss of argon ions.

Rutherford backscattering spectrometry (RBS) was performed to understand and quantify ion-beam induced amorphization and recrystallization. PARAS facility at Inter University Accelerator Center, New Delhi has been employed in random and channeling geometry for RBS measurements. Channeling experiments were carried out with ion beam parallel to (111) axis^30–32^ and corresponding image and angle scan have been collected by NEC’s RC43 Software. The damage profiles were simulated from the RBS/C spectra using the code DICADA, which is based on the continuous model of dechanneling^14^.

The structural alterations resulting from ion beam induced amorphization and subsequent annealing process was studied as a function of off-normal incidence of argon ions by Raman spectroscopy. Micro-Raman scattering measurements were performed using STR 500 Confocal µ-Raman spectrometer for Doide-pumped laser tuned at 532 nm in backscattering geometry. Here, we have recorded Raman spectra at 4–5 different places on one sample and each time, focusing was done separately before recording the spectra. It was found that the variation in the Raman intensity arising from different areas of the film is minimal.

## Results and discussion

### RBS/C investigations of as amorphized and recrystallized Si(111) surfaces

**Figure 1:**
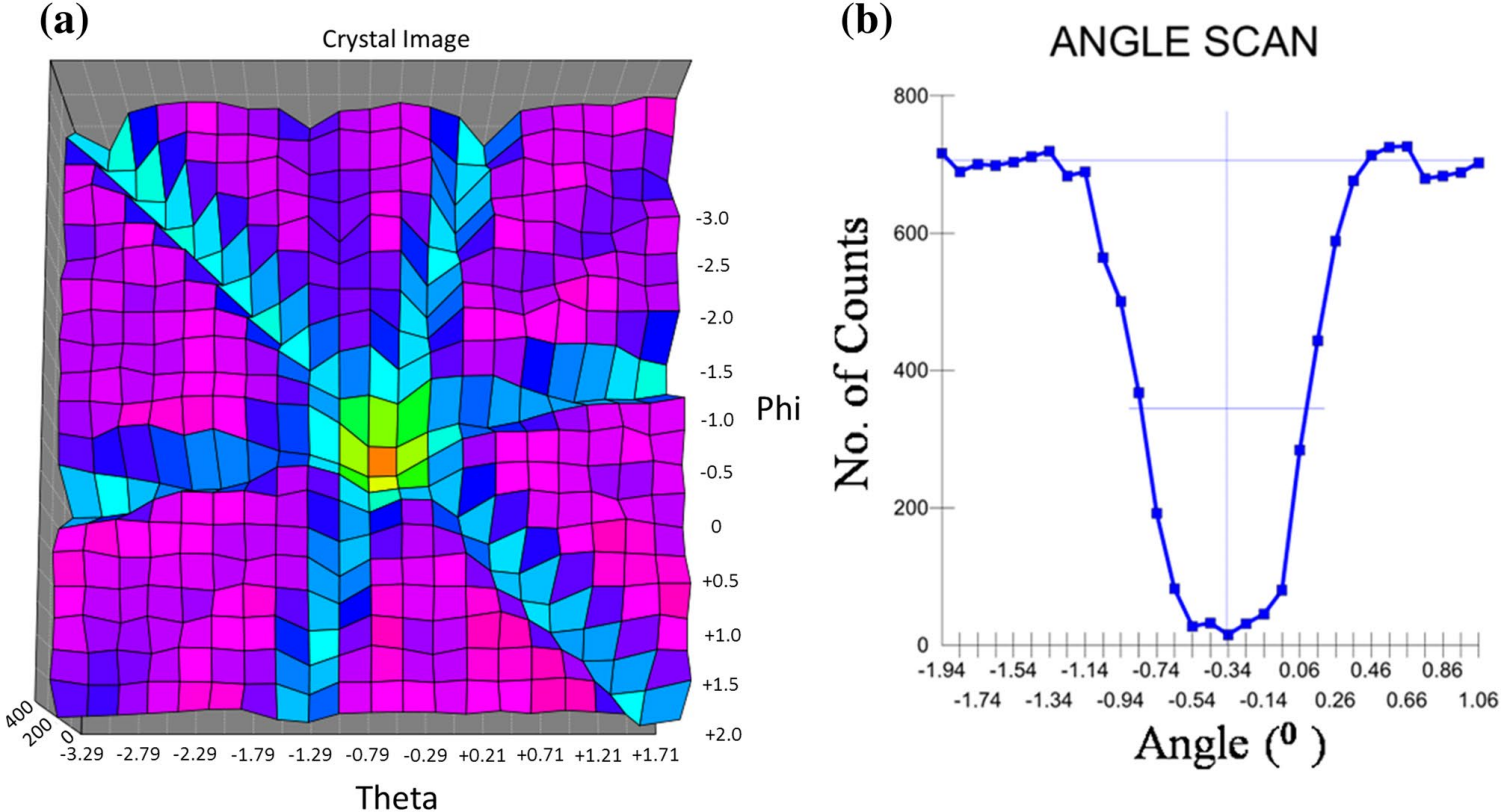
(**a**) Image scan for crystalline-Si(111). (**b**) Angle scan for crystalline-Si(111).

Rutherford Backscattering Spectrometry in channeling geometry (RBS/C) allows qualitative identification of the crystal orientations in single crystal lattice^30,32^. The identification of the channels in Si(111) (image and angle scan) carried out in RBS/C employing NEC’s RC43 Software is presented in Fig. 1a,b.

It is clear from Fig. 1b that ψ_1/2_ is 0.38°, calculated from half the yield level half way between the minimum and random level^32^. This small value of ψ_1/2_ clearly illustrates that the scanned ⟨111⟩ is the principal crystal axis in Si wafers used in the present studies.

**Figure 2:**
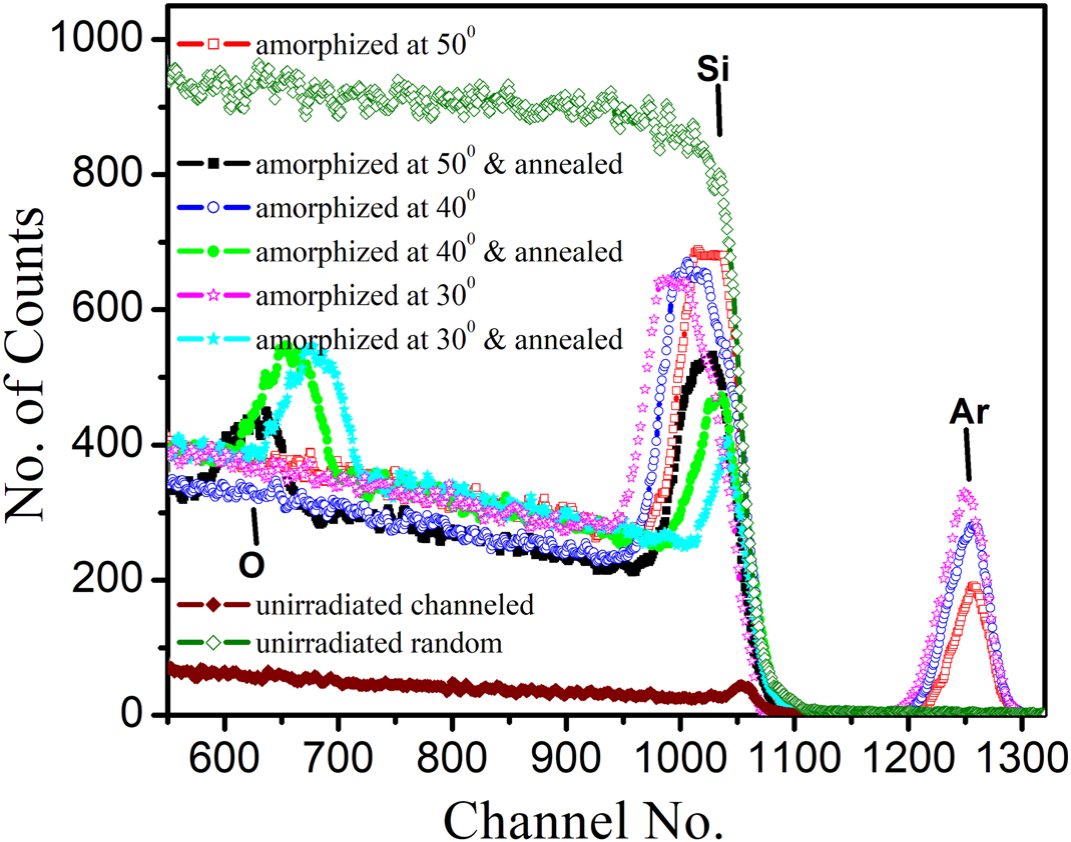
RBS random and RBS/C spectra along the ⟨111⟩ axis in un-irradiated, amorphized and annealed Si(111) samples at 1,073 K.

Further, RBS in random and channeling geometry qualitatively analyze the crystalline, amorphous and recrystallization content as a function of depth in these samples. A set of RBS/C spectra recorded for amorphized and recrystallized Si(111) specimens are presented in Fig. 2. For comparison, we have plotted the RBS spectra in random and ⟨111⟩ axis of the un-irradiated and unannealed crystalline Si (c-Si) in Fig. 2.

In Fig. 2, the spectrum taken in the random direction displays edge at channel no. 1059 (energy value of 1.136 MeV) corresponding to the backscattering of analyzing particles from the un-irradiated silicon^14,30^. The spectrum taken in the (111)-axial direction in the irradiated and annealed samples displays the same edge of silicon but with a lower backscattering yield due to channeling effect. After irradiation to a dose of 3 × 10^17^ Ar^+^cm^−2^, the amorphized samples (Fig. 2) displays damage peak corresponding to amorphous silicon and an additional peak at energy value of ~ channel no. 1249–1269. Using RUMP, this peak has been identified as of argon. Moreover, in case of amorphized and annealed samples, there occurs similar damage peak and an additional peak at ~ channel no. 629–680 belonging to oxygen^30^.

For un-irradiated and unannealed Si(111), the backscattering yield of the amorphous peak along the ⟨111⟩ axis is found to be only 5%, which signifies the high crystalline quality of the surface layers^31^.

After amorphization at different oblique incidences of 50°, 40° and 30°, the presence of damage peak in RBS/C spectra at channel no. 1059 along the ⟨111⟩ axis reveal the formation of amorphous layer due to argon beam induced disordering in the silicon lattice^31^. The channeled backscattering yield corresponding to this damage peak decreases with decrease in oblique incidence (Fig. 2). The values of backscattering yield for un-irradiated, irradiated and annealed Si(111) have been summarized in Table 1.

**Table 1:** Backscattering yield values for un-irradiated, irradiated and annealed Si(111) samples as a function of ion incidence.

Specimen	Backscattering yield
**Un-irradiated Si(111)**	
Random	849.85 ± 6.71
Channeled	69.85 ± 0.21
**As amorphized with 80 keV Ar**^**+**^** ions at off-normal incidence**	
50°	685.85 ± 4.23
40°	670.45 ± 3.82
30°	658.85 ± 2.64
**Annealed at 1,073 K**	
50°	536.12 ± 2.14
40°	480.76 ± 1.94
30°	435.45 ± 1.30

From Table 1, it is clear that the backscattering yield in the amorphized and annealed samples decreases with decrease in oblique incidence. Based on these values the amorphous content in damage peak has been estimated to be 79%, 77% and 75.4% for oblique incidences of 50°, 40° and 30°. Hence, irradiated Si(111) samples are not completely amorphized under oblique Ar^+^ sputtering. However, Turos et al.^24^ reported complete amorphization in Si(111) with 2 × 10^15^ Ne ions cm^−2^ at 60 keV for normal incidence. Also, Nakata et al^4,12^ have observed complete amorphization in Si(100) and Si(110) with 50 keV As^+^ at a fluence of 5 × 10^15^ ions cm^−2^ at normal incidence.

After the annealing of the amorphized samples, a good recovery of the damage has been observed for all samples (Fig. 2). We note that the channeled yield reduces to 60%, 54% and 48% for recrystallized Si(111) specimens at oblique incidences of 50°, 40° and 30°. Thus, RBS channeling experiment demonstrated that crystalline behavior restores partially after recrystallization. In contrast to our finding, Turos et al.^24^ have observed complete recovery in amorphized Si samples at annealing temperature of 800 °C.

The presence of additional oxygen peak in recrystallized samples (Fig. 2) is logically due to annealing process in the oxygen atmosphere. Significant shift in the oxygen peak position towards the surface with decrease in oblique incidence has been observed. Concerning the argon ions, the implanted Ar profile in-diffuses with decrease in oblique incidence after amorphization at different oblique incidences of 50°, 40° and 30°. But, it is important to note that implanted Ar escaped out completely during the annealing treatment.

**Figure 3:**
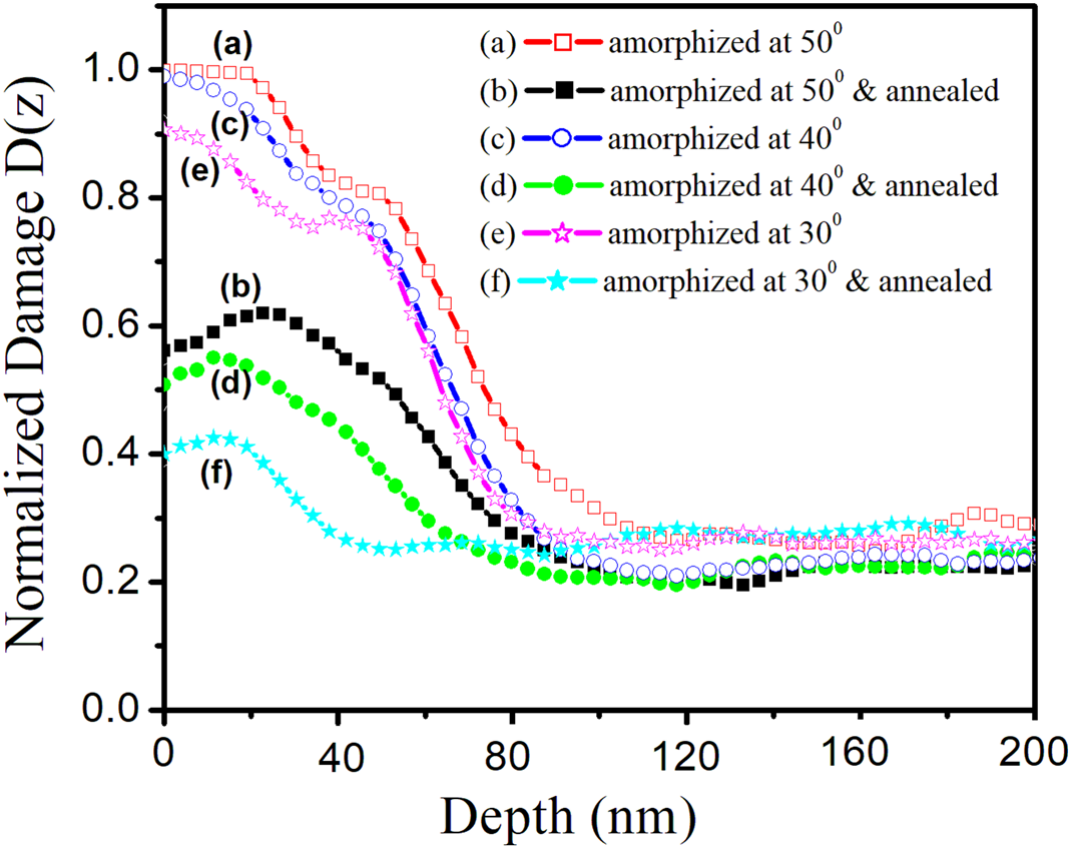
DICADA^14^ simulated depth distribution of normalized damage D(z) in amorphized and annealed Si(111) samples at 1,073 K.

To study in detail the amorphization and recrystallization behavior, we present in Fig. 3 the damage profiles D(z) as a function of penetration depth of incoming ions for different angle of incidences. These profiles have been simulated from the RBS/C measurements using DICADA code^14^.

The thickness of the amorphous layer produced under oblique argon ion irradiation and recrystallized layer under annealing treatment has been determined by estimating the full width at half maxima (FWHM) of these damage profiles. The width of the amorphous layer in Ar^+^-amorphized samples at oblique incidences of 50°, 40° and 30° comes out to be 80 nm, 73 nm and 69 nm; evidencing the formation of amorphous layers of variable thickness. It is very much clear that complete amorphization has not been attained in our present case. We note that maximum of these profiles lies in the surface region as clear from Fig. 3a,c,e. As a consequence, only surface amorphization is induced by oblique argon ion sputtering (Fig. 3a,c,e). Due to this surface amorphization, asymmetry is present in these damage profiles. One noticeable feature is that these damage profiles exhibit similar behavior towards the end of damage region and saturates in the near surface region. These observations are further confirmed by structural studies as discussed later.

In case of annealing treatment at 1,073 K, the width of the damage profile starts decreasing significantly (Fig. 3b,d,f). This emphasizes that the amorphized layer thickness as well as damage reduces drastically for the recrystallized specimens. With decrease in oblique incidence, D(z) reduces drastically which indicates increasing recrystallization as a function of oblique incidence. This decrease is found to be 42% of the total damage at oblique incidence of 30°, resulting in recrystallized layer of thickness 32 nm. Surprisingly, complete recrystallization could not be attained upon thermal annealing at 1,073 K. This can be attributed to the agglomeration of isolated point defects into extended defect zones^11,18^, thereby, making difficulty in annealing even at a temperature of 1,073 K.

We have observed out diffusion of oxygen with decrease in oblique incidence from 50° to 30° (Fig. 2). This shift in O peak position is attributed to depth varying amorphization induced by oblique argon ion sputtering. The amorphous layer thickness is found to be 80, 73 and 69 nm for oblique incidences of 50°, 40° and 30° (Fig. 3). Due to the decrease in amorphous layer thickness with oblique incidence from 50° to 30°, we observed out diffusion in oxygen during annealing treatment.

One can clearly see that though the width of these damage profiles decreases but they are still asymmetric in nature. As a consequence, the maximum of damage distribution shifts towards near surface region as compared to amorphized samples (Fig. 3). This indicates the formation of buried amorphous layer in these as amorphized and annealed Si(111) samples.

**Figure 4:**
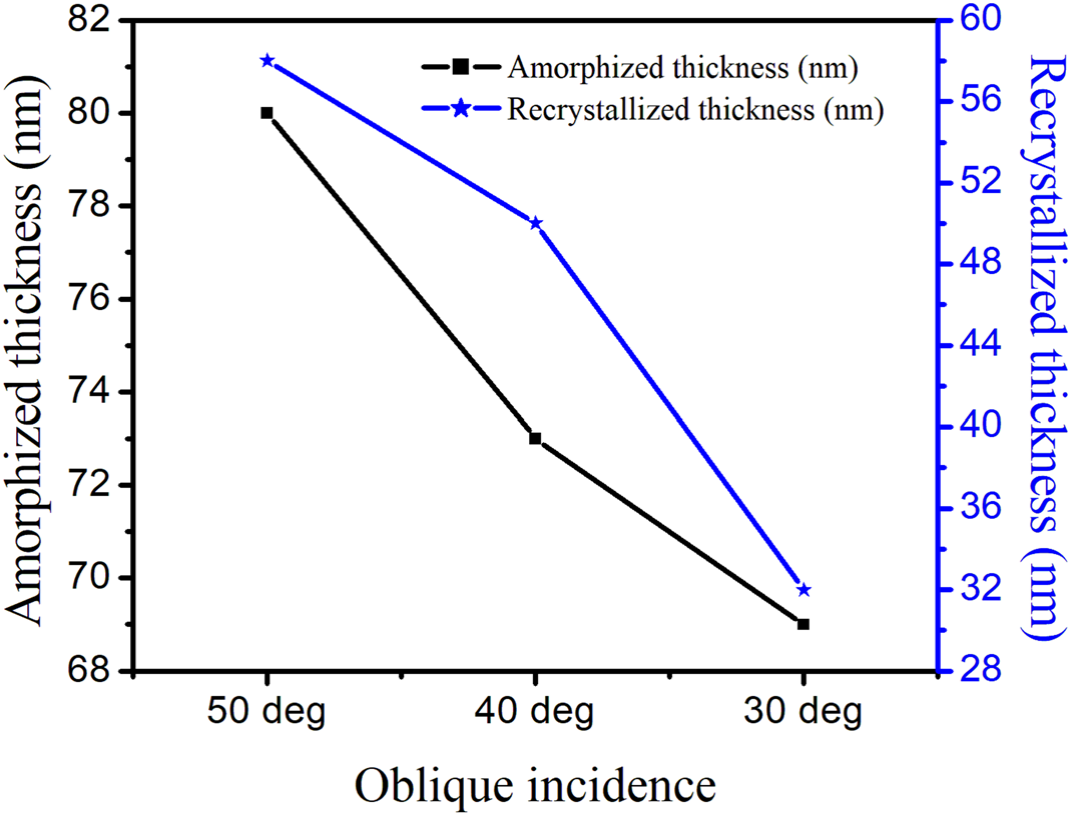
Plots showing the dependence of the amorphized and recrystallized thickness on the oblique incidence.

The amorphous and recrystallized layer thickness as a function of angle of incidence is presented in Fig. 4.

Figure 4 reveals that both the amorphous as well as recrystallized layer thickness reduce with decrease in oblique incidence; evidencing direct dependence on the angle of incident argon ions.

**Figure 5:**
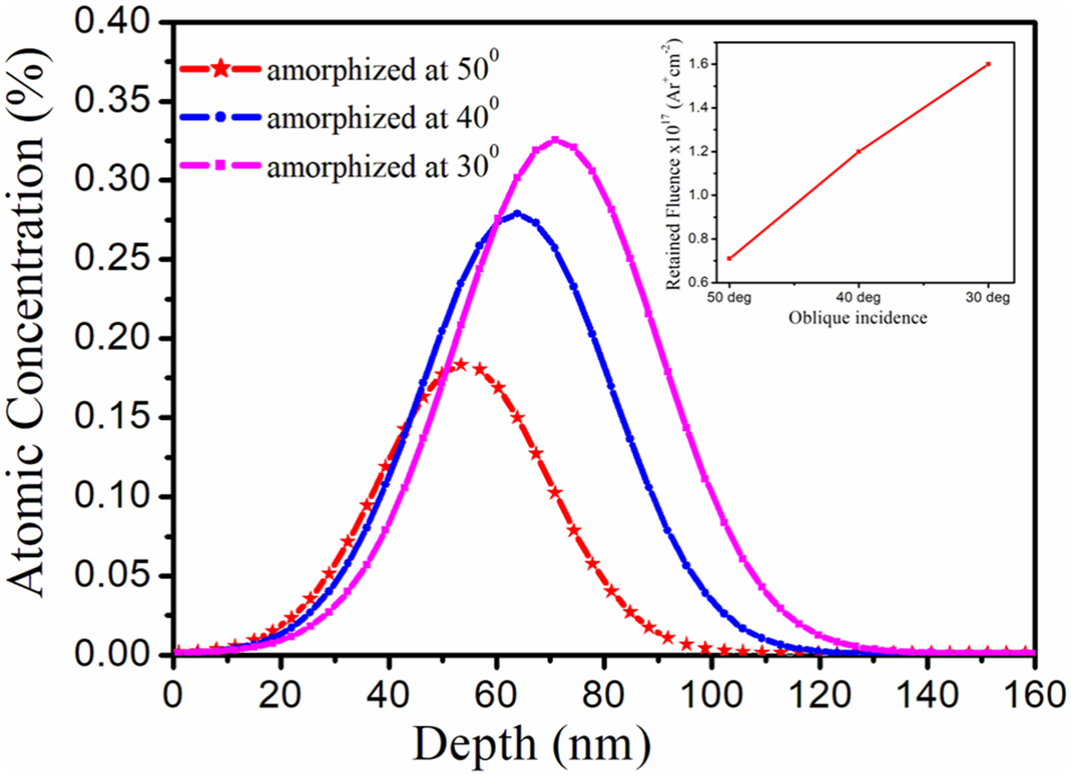
RBS random and RBS/C spectra along the ⟨111⟩ axis in amorphized Si(111) samples.

The simulated distribution of argon in the Si(111) lattice with penetration depth^30^ for different oblique incidences from RBS/C spectra after amorphization at different off-normal incidences has been plotted in Fig. 5.

Figure 5 reveals Gaussian profile of argon distribution in the Si(111) lattice as a function of penetration depth for all the amorphized specimens. Interestingly, this depth distribution of Ar enhances into the bulk Si(111); hereby evidencing in-diffusion of argon with decrease in oblique incidence. In addition, maximum surface concentration of Ar is found at lowest oblique incidence of 30° thus reaching 0.35 at.%. The calculated fluence of Ar ions differ by nearly 7% from the original fluence (3 × 10^17^ Ar^+^cm^−2^) used in sputtering experiment, as shown in the inset of Fig. 5. The enhanced in-diffusion of Ar into Si(111) lattice can be accounted in terms of oblique argon ion irradiation induced surface amorphization and defects under high flux. The projected range (R_P_) and standard deviation (ΔR_P_) are estimated to 54 ± 26 nm, 64 ± 32 nm and 75 ± 34 nm for different off-normal incidences of 50°, 40° and 30°. These values of range compare well with TRIM simulation results of 55 ± 28 nm, 65 ± 30 nm and 77 ± 31 nm^33^.

Our RBS/C results reveal that irradiated Si(111) are not completely amorphized under 80 keV argon ion sputtering at oblique incidence of 50°, 40° and 30° for a fluence of 3 × 10^17^ Ar^+^cm^−2^. Upon annealing at 800 °C, partial recrystallization has been observed in these pre-amorphized samples. This can be due to highest atomic density in Si(111) in comparison to other planar orientations of Si. Nakata et al.^4,12^ have also reported that crystallized thickness of Si(100) was approximately two times than that for Si(111). In addition, Nakata et al.^4,12^ have observed complete amorphization in Si(100) and Si(110) with 50 keV As^+^ at a fluence of 1 × 10^15^ ions cm^−2^ at normal incidence but Turos et al.^24^ reported complete amorphization in Si(111) at 60 keV for normal incidence but with double fluence of 2 × 10^15^ Ne ions cm^−2^.

### Raman investigations of as amorphized and recrystallized Si(111) surfaces

**Figure 6:**
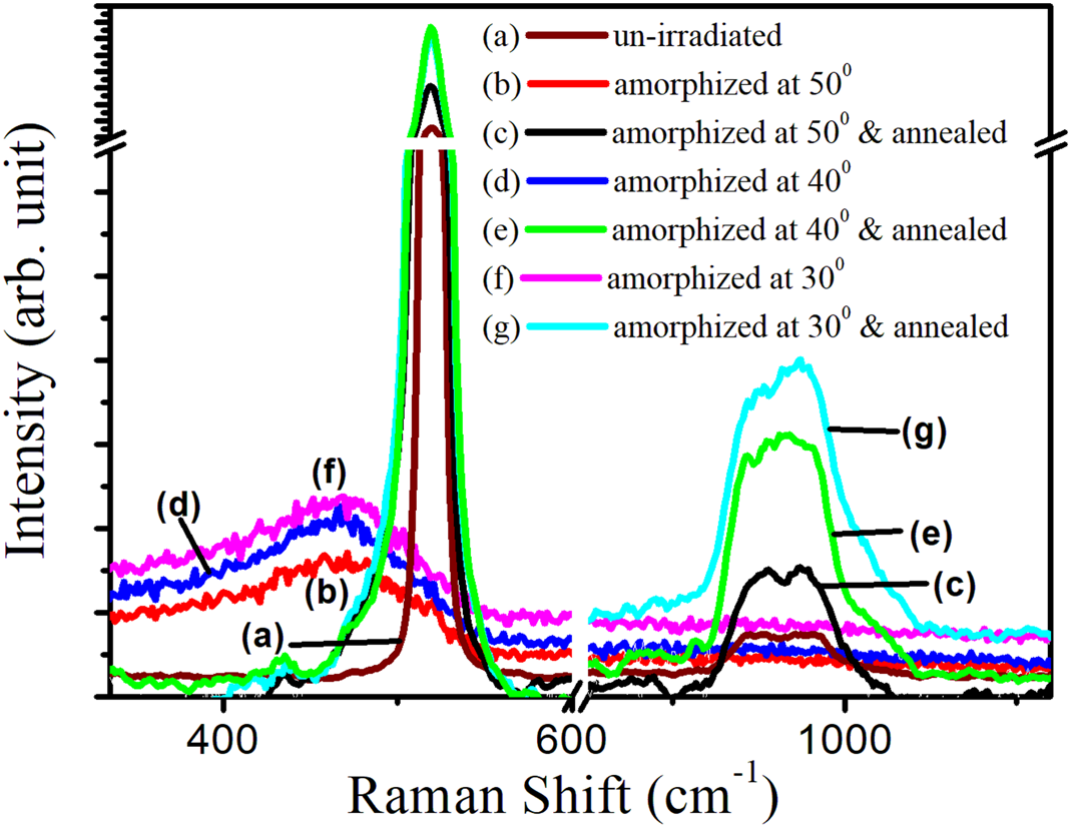
Raman spectra corresponding to Ar^+^ amorphized and annealed samples as a function of off-normal incidence.

Raman spectra of amorphized and recrystallized Si samples at different off-normal incidences of 30°, 40° and 50° have been presented in Fig. 6. The spectrum of un-irradiated Si(111) is also shown for comparison. For un-irradiated Si(111), the Raman first order spectrum consists of one strong crystalline peak (c-Si) at 521 cm^−1^ and broad second order peak in the range 920 to 1,030 cm^−1^ (Fig. 6a). These peaks are associated with the triply degenerate transverse optical phonon (1TO and 2TO) corresponding to zone center^34^.

For oblique Ar^+^ amorphized Si(111) samples (Fig. 6b,d,f), the spectra consists of a broad amorphous band (a-Si), being centered at (450 ± 3) cm^−1^. This band is ascribed to the amorphous silicon (a-Si) having transverse optic TO mode^35–39^. The appearance of a-Si peak (Fig. 6b,c,d) at the expense of triply degenerate transverse optical phonon (1TO and 2TO) peaks (Fig. 6a) for the as amorphized samples show the amorphization in sub-surface region only. This is also reflected in the RBS/C spectra shown in Fig. 3.

After annealing at 1,073 K, apparent sign of recrystallization can be seen from the Raman spectra (Fig. 6c,e,g). With decrease in oblique incidence, the peak related to the crystalline component increases and becomes more symmetric; which indicates increasing recrystallization with decrease in oblique incidence. However, the presence of small amorphous content in these samples results in a tail in this c-Si peak. This clearly demonstrates that the decreasing contribution of amorphous phase accounts for the corresponding increase of crystalline phase.

However, the peak related to c-2TO phonon vibrations is found to be becoming broader and symmetric with decrease in oblique incidence. This strongly emphasizes the dependence of recrystallization behavior on the depth varying surface amorphization induced by obliquely incident argon ions. These findings corroborate the RBS/C results showing the partial recrystallization after annealing treatment.

Besides recovery by annealing treatment, some stress still remains in the recrystallized Si(111) samples. This remnant stress has been signified by shifting of the Raman peak in these recrystallized specimens towards the lower wave numbers in comparison to un-irradiated c-Si, which is stress free^18,35–39^. The magnitude of this stress has been estimated^39^ using the relation:

$$\sigma\,(\text{MPa})=250\Delta \omega ({\text{cm}}^{-1})$$

where σ is the magnitude of the stress, $\Delta \omega$ is the peak shift. From above equation $\Delta \omega$ has been calculated and is shown in Table 2 for amorphized and annealed specimens.

**Table 2:** Raman peak position, line-width variation and calculated stress for amorphized and annealed Si(111) specimens.

Specimen	Raman peak position (cm^−1^)	FWHM (cm^−1^)	Stress (GPa)
Un-irradiated Si(111)	520	9 ± 0.2	0
**As amorphized with 80 keV Ar**^**+**^** ions at off-normal incidence**			
50°	469	140 ± 5	12.75
40°	465	146 ± 7	13.75
30°	462	150 ± 8	14.50
**Annealed at 1,073 K**			
50°	515	12 ± 0.4	1.00
40°	517	11 ± 0.5	0.50
30°	519	10 ± 0.6	0.25

It can be concluded from Table 2 that stress is increased with decrease in oblique incidence for amorphized samples while this tensile stress decreases for recrystallized samples. Furthermore, the stress is not completely removed even after annealing treatment at 1,073 K.

### Mutual correlation

**Figure 7:**
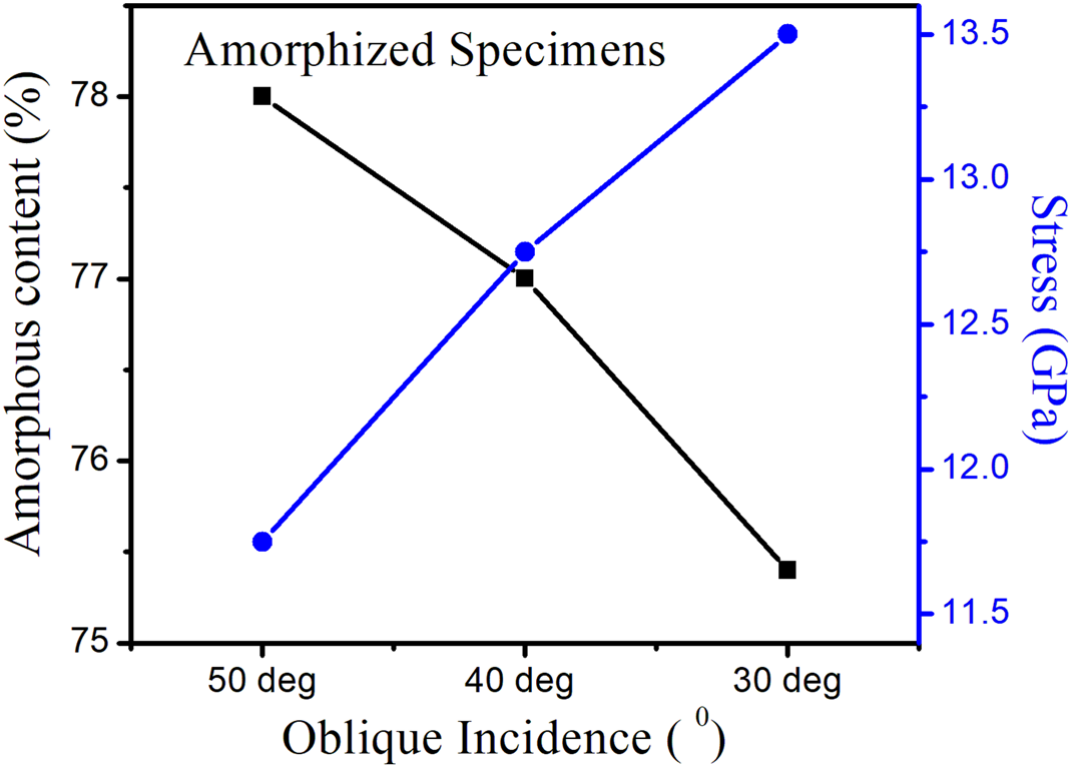
Amorphous content and relative stress for amorphized samples as a function of off-normal incidence.

It is clear from Fig. 7 that the decrease in amorphous content with decreasing off-normal incidence results in increased stress in the irradiated samples.

After annealing of these amorphized samples, the degree of amorphous content and stress reduces significantly. We found that decrease in amorphous content with decreasing off-normal incidence results in decreased stress in the recrystallized samples contrary to the relative increase in the stress in amorphized samples.

**Figure 8:**
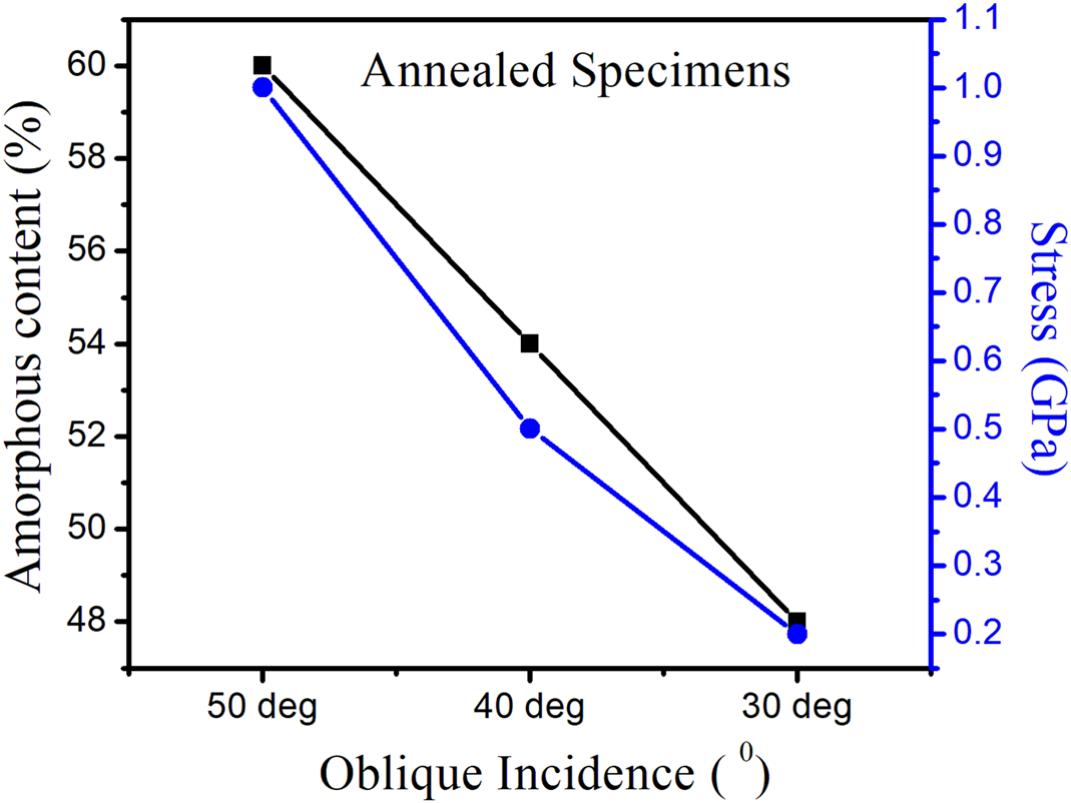
Amorphous content and relative stress for annealed samples as a function of off-normal incidence.

Hence, it can be inferred from Figs. 7 and 8 that depth dependent surface amorphization and hence recrystallization is due to oblique incidence induced sputtering and results in angle dependent tensile stress in the samples.

The observed damage profiles of silicon in irradiated and annealed Si(111) samples relies on nuclear energy loss dominated sputtering process and defect generation^40–42^. The present experiment has been carried out at oblique incidence of 50° and therein, sputtering effect comes into picture. So, to quantify its role in amorphization and recrystallization, we have estimated the sputtering yield (SY) and hence the energy transferred by incoming argon ions to recoiling Si atoms at these oblique incidences in Si(111) lattice.

Table 3 reveals that the sputtering yield and energy transferred to recoiling Si atoms is highest for 50° off normal incidence of irradiation. This shows that for this oblique incidence, the incoming argon ions have appropriate energy and momentum to eject the Si atoms lying in the vicinity of surface rather than imparting its energy in the sub-surface layers. Therefore, silicon atoms lying close to the surface gain sufficient energy to leave the surface and get sputtered out^40–42^. Table 3 further depicts that both these parameters decreases with decrease in angle of incidence. Hence, energetic argon ions start depositing their energy in the near surface region in addition to surface layers and results in increase in depth profiles of relative disorder of Si with decrease in oblique incidences.

**Table 3:** Magnitude of SY and energy transferred to recoils for different angle of incidences.

Angle of incidence (°)	Sputtering yield (Si atoms/Ar^+^ ion)	Energy transferred to recoils
30	0.96	70
40	1.35	80
50	2.16	100

Secondly, the peak position of disorder profile depends on the backscattering probability of He-ion versus the evolution of defects and microstructure induced as a result of obliquely incident argon ions. Hence, variations in irradiation-induced defects and isolated amorphous zones at different oblique incidences affect the energy of backscattered He-ion and are responsible for the depth shift of the disorder peak^41,42^.

Further, the recrystallization kinetics can be efficiently understood through the number of sputtered ions and vacancies created by local the temperature rise due to the nuclear energy loss. In partially disordered Si samples, where isolated regions of amorphous Si are embedded inside c-Si, annealing treatment at 1,073 K leads to recrystallization. This may be due to the synergic effect of both the sputtered ions generated by sputtering process and nuclear energy loss produced interstitial pairs at the crystalline/amorphous (c/a) interface. Therefore, the recrystallization dynamics is governed by the rate of production of defects as well as interstitials at the c/a interface and lying close to it. So, sputtering induced sputtered ions (Ar and Si) and the annealing treatment induced additional vacancies reach the interface and could help to enhance the re-growth process.

## Conclusions

Present studies summarize the investigations of recrystallization of pre-amorphized Si by annealing treatment. Results demonstrate that the damage retained in the substrate is highly dependent upon oblique incidence of argon ions. The depth profiles reveal the out diffusion of Ar with increase in oblique incidence for amorphized samples. The micro-Raman indicates the remnant stress even after the annealing treatment at temperature of 1,073 K. The re-growth rate dynamics is governed by the production and migration of irradiation induced defects and vacancies near to crystalline/amorphous interface due to the dominance of sputtering phenomenon. This study reveals that oblique ion beam incidences induced sputtering process followed by annealing treatment results in depth varying recrystallization of pre-amorphized Si(111) layers.

## Supplementary information

Supplementary Information.
