# Supplementary material for: Nano-scale depth-varying recrystallization of oblique Ar+ sputtered Si(111) layers
Source: Sci Rep. 2020 Jul 17;10:11905. doi: 10.1038/s41598-020-68873-8 (PMC7367853; doi:10.1038/s41598-020-68873-8)
Supplement: Supplementary file 1 — Supplementary Information. [file 41598_2020_68873_MOESM1_ESM.docx]

**Nano-scale depth-varying recrystallization of oblique Ar^+^ sputtered Si(111) layers**

Divya Gupta^1^, G. R. Umapathy^2^, Rahul Singhal^3^, Sunil Ojha^2^ and Sanjeev Aggarwal^1,a^

*^1^Department of Physics, Kurukshetra University, Kurukshetra-136119, India*

*^2^Inter University Accelerator Center, Aruna Asaf Ali Marg, New Delhi-110067, India*

*^3^Malviya National Institute of Technology (MNIT), Jaipur, Rajasthan-302017, India*

^a^*E-mail address:* [guptadivvi@gmail.com](mailto:guptadivvi@gmail.com); [saggarwal@kuk.ac.in](mailto:saggarwal@kuk.ac.in)

**Supplement-1: Interpretation of image scan of RBS in figure 1**

Rutherford Backscattering spectrometry in channeling mode (RBS/C) is a versatile tool for the identification of crystal planes in single crystal lattice. When the probing He particles are directed towards the single crystal along one of its major axes or planes, most of incident particles escape close collision events. This absence of close collision events provides a direct method for determining the orientation of a crystal defined by its major axes and planes with respect to the incident beam direction and forms the basis of RBS channeling.

This is achieved by searching for the orientation of the crystal that yields the minimum counts of close collision events under constant bombardment by the probing He particles. This search is usually performed with a series of angular scans. An angular scan consists of observations of the number of close collision events when the crystalline lattice is bombarded with an appropriate dose of ions at a fixed energy incident at each of a sequence of directions perpendicular to one crystalline axis. Besides determining crystal orientation, such angular scans can also be used to probe the details of the lattice structures, especially deviations from the ideal. RBS/C is widely used for the location of impurity atoms, lattice disorder, and composition of amorphous surface layer.

To observe the nanoscale depth varying recrystallization of oblique Ar^+^ sputtered Si(111) layers, RBS and channeling experiment were performed.

The random measurement of samples backscattered He^+^ ions energy is recorded for plane inclined at 7^0^ to avoid channeling in Si. Then to find channeling axis, image was scanned in the plane of XY from -3.5 to 3.5 in steps of 0.1 tilt angle in both axis (starting ϴ and Ф at zero position). Here at each interval, back scattered spectra of ions was collected and colored pattern displayed on screen with relative grading of colors from pink (for max He^+^ ions collected ) to orange (for min He^+^ ions collected). The orange box in Fig. 1.1 is the area of minimum back scattered zone. Corresponding to this Theta and phi angels are noted and for more accuracy in reading ϴ and Ф we perform angle scan individually. Fixing these values in the goniometer along with ϴ and Ф position, channeled spectra is recorded.

The primary step for channeling experiment is crystal alignment to perpendicular to the beam direction. The 4 axis goniometer is capable to move in ‘x’ and ‘y’ direction and tilt in ‘theta’ and ‘phi’ direction. The ‘x’ and ‘y’ position is fixed so that beam is incident on the center of the sample. The Image scan window in NEC’s RC43 Software allows prefixing theta and phi scan in integrated steps. The image scan is assigned to 20x20 theta to Phi squares. The Automated RC43 records backscattered data in each interval in minimum negative value to max value. The rocking continues to form the first row to the last row of the last interval. On the basis of data collected RC43 allotting different color form max at Red -100 to brown -0.


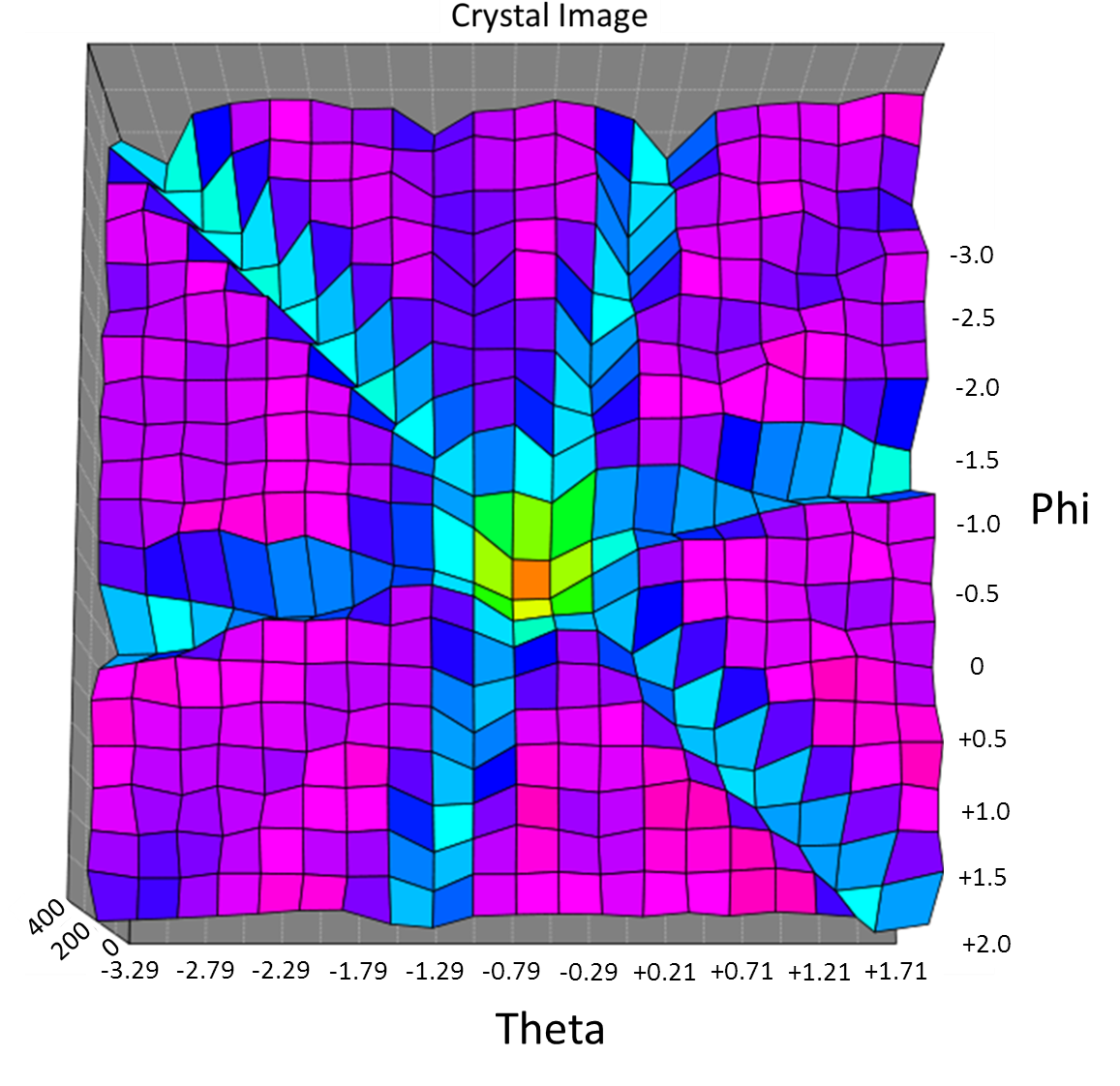


**Figure 1.1** Image scan of Si(111)

In general, angular scan is obtained by measuring the scattering yield in a certain energy window or depth interval as a function of the angle between the ion beam and the crystal direction. The width of the angular scan is characterized by the half angle ψ_1/2_ for an ion to become channeled in a major axial or planar direction. A typical angular scan near a principal crystalline axis shows a dip in a plot of the yield of close collision events versus the incident direction of the ion beam relative to the crystal axes. An ideal angular scan would be performed in a plane containing the principal crystalline axis of interest. The direction of the ion beam with the minimum yield is then identified with the principal crystal axis. The half-width of the dip at a yield level half way between the minimum and random level, which is the yield at most other directions far enough away from the channeling direction, is identified with the half-angle, ψ_1/2_ for channeling. This makes ion-channeling in combination with RBS an excellent tool for analyzing lattice deformations in crystalline structures.

Angular scan for Si(111) substrate is shown in Fig. 1.2 along with the half angle. In our case, ψ_1/2_ is 0.38^0^.

**
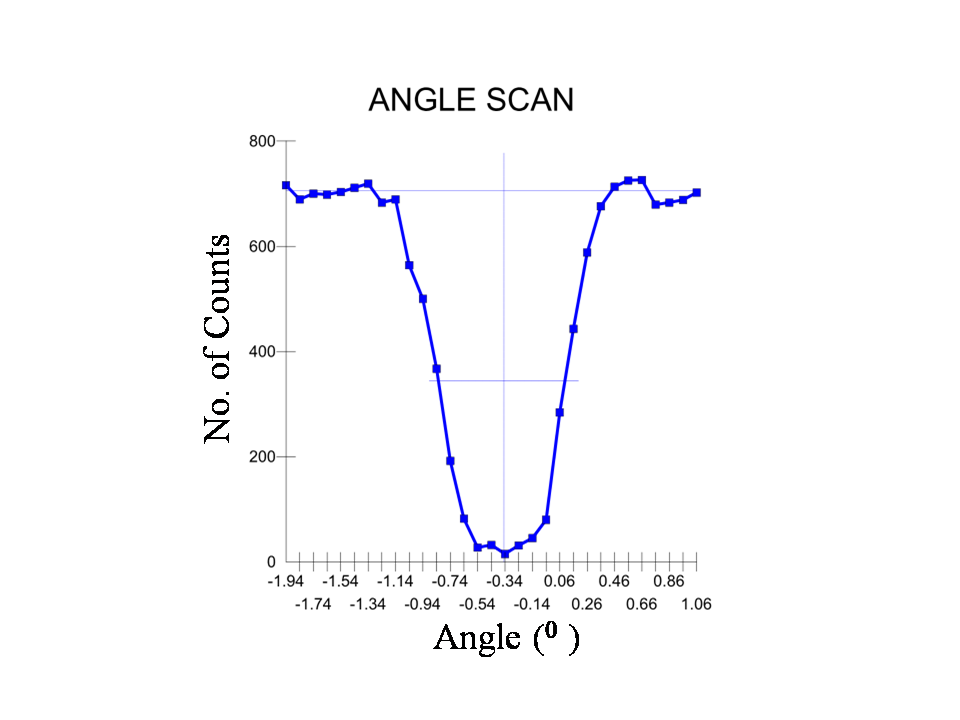
**

**Figure 1.2** Angle scan of Si(111)

**Supplement-2: The methodology for Ar profile measurement**

This article describe how the energy *E* of the detected particle is related to the depth *x* at which the backscattering event occurs in a mono-isotopic sample (Fig. 1).


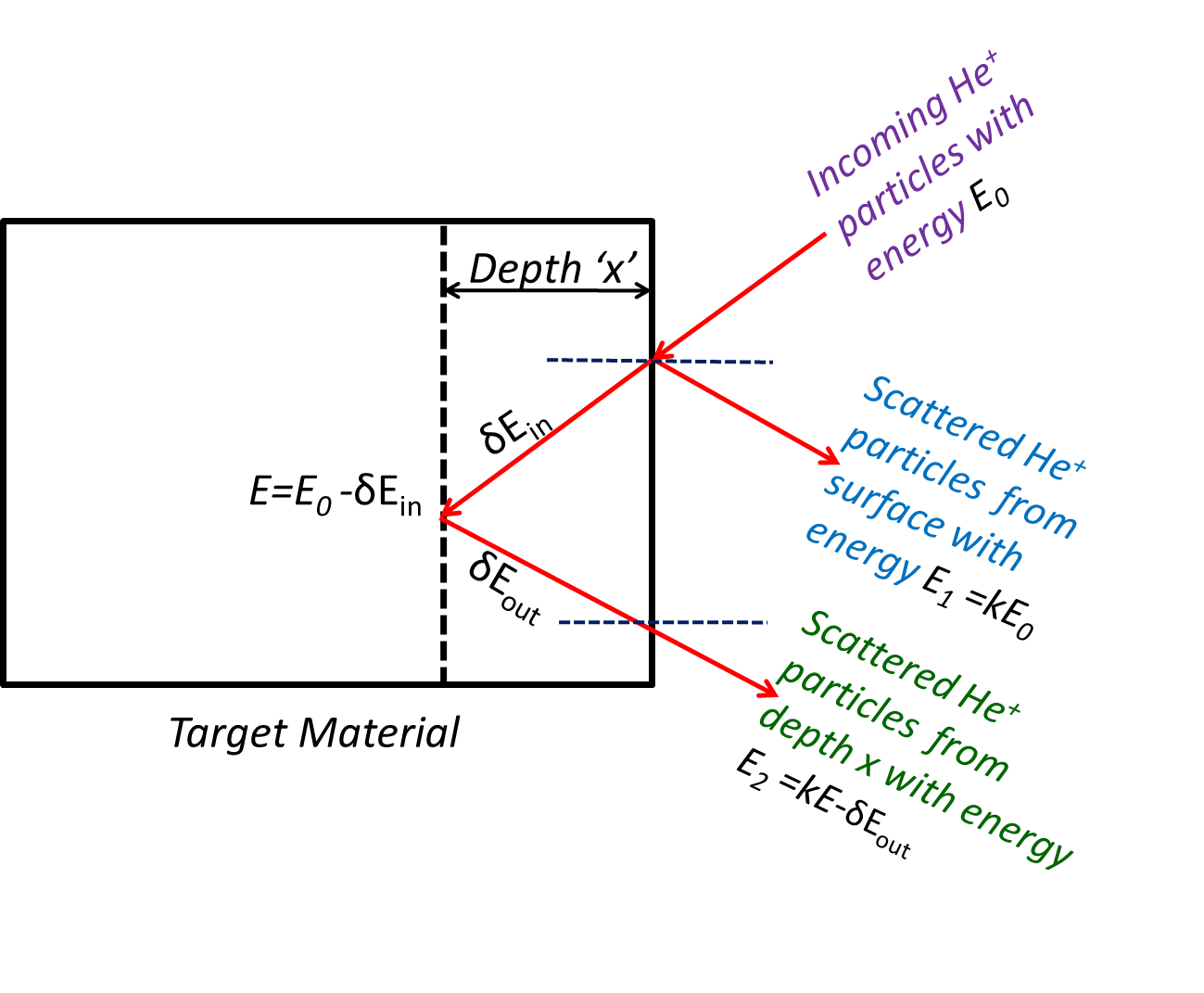


**Figure 1.3** Backscattering depth profile geometry

Let the energy of the incident particles is *E_0_* and the energy of the particle before scattering at a depth *x* is *E*. The energy of a particle scattered from the surface is *E_1_* (= *KE_0_*) and that from a scattering at depth *x* is *E_2_*.

The incident particle, the scattered particle and the normal to the sample surface are all contained in one plane, so that the scattering angle in the laboratory frame of reference is given by *θ* = *180°* - *θ_1_* – *θ_2_*, where *θ_1_* and *θ_2_* are the angles between the sample normal and the direction of the incident beam and of the scattered particles, respectively.

The energy *E* and the length *x*/*Cos* *ϑ_1_* of the incident path is related by

$$\frac{x}{Cos\theta_{1}}=-\int_{E_{0}}^{E} dE/\left(dE/dx\right)$$

where the negative sign arises because *E* is smaller than *E_0_* and *dE/dx* is taken as a positive quantity.

The energy of the scattered particle from depth *x* is *KE* and the path length *x*/*Cos* *ϑ_2_* of the outward path is given by

$$\frac{x}{Cos\theta_{2}}=-\int_{KE}^{E_{2}} dE/\left(dE/dx\right)$$

The difference *E_0_ — E* is the energy loss along the inward path and *KE* — *E_2_* is the energy loss along the outward path. The energy *E* before scattering is not an experimentally accessible quantity, but *E_0_* and *E_2_* are. So one needs to find *x* in terms of *E_0_* and *E_2_*.

Here, we assumed a constant value for *dE/dx* along the inward and outward paths, the two integrals in above eqns. reduce to

$$E=E_{0}-\frac{x}{Cos\theta_{1}}\left(\frac{dE}{dx}\right)_{in}$$

$$E_{2}=KE-\frac{x}{Cos\theta_{2}}\left(\frac{dE}{dx}\right)_{out}$$

The subscripts “in” and “out” refer to the constant values of *dE/dx* along the inward and outward path.

Eliminating E from these equations, we get

$$KE_{0}-E_{2}=\left[\frac{K}{Cos\theta_{1}}\left(\frac{dE}{dx}\right)_{in}+\frac{1}{Cos\theta_{2}}\left(\frac{dE}{dx}\right)_{out}\right]x$$

The energy *KE_0_* = *E_1_* is the edge of the backscattering spectrum and corresponds to the energy of particles scattered from atoms at the surface of the target. The energy *E_2_* is the measured value of a particle scattered from an atom at depth *x*. If one introduces the symbol *ΔE* for the energy difference between *E_2_* and *KE_0_*

$$\Delta E=KE_{0}-E_{2}=\left[\frac{K}{Cos\theta_{1}}\left(\frac{dE}{dx}\right)_{in}+\frac{1}{Cos\theta_{2}}\left(\frac{dE}{dx}\right)_{out}\right]x$$

$$\Delta E=\left[S\right]x$$

$S=\left[\frac{K}{Cos\theta_{1}}\left(\frac{dE}{dx}\right)_{in}+\frac{1}{Cos\theta_{2}}\left(\frac{dE}{dx}\right)_{out}\right]$is the energy loss factor.

$$\Delta E=\left[\varepsilon\right]Nx$$

The assumption of constant values for *dE/dx* or *Ɛ* along each track thus leads to a linear relationship between the energy *ΔE* below the edge *KE_0_* and the depth at which scattering occurs. Thus, we have assigned a linear depth scale to the energy axis.

**Figure Legends**

**Figure 1.1** Image scan of Si(111)

**Figure 1.2** Angle scan of Si(111)

**Figure 1.3** Backscattering depth profile geometry
